# Antibiotic use and clinical outcomes in the acute setting under management by an infectious diseases acute physician versus other clinical teams: a cohort study

**DOI:** 10.1136/bmjopen-2015-010969

**Published:** 2016-08-23

**Authors:** Nicola JK Fawcett, Nicola Jones, T Phuong Quan, Vikash Mistry, Derrick Crook, Tim Peto, A Sarah Walker

**Affiliations:** 1 Nuffield Department of Medicine, University of Oxford, Oxford, UK; 2 Department of Acute/General Medicine, Oxford University Hospitals NHS Foundation Trust, Oxford, UK; 3 Nuffield Department of Medicine, NIHR Health Protection Unit in Healthcare Associated Infections and Antimicrobial Resistance, University of Oxford, NIHR Oxford Biomedical Research Centre, Oxford, UK

**Keywords:** INFECTIOUS DISEASES, GENERAL MEDICINE (see Internal Medicine), INTERNAL MEDICINE, MICROBIOLOGY

## Abstract

**Objectives:**

To assess the magnitude of difference in antibiotic use between clinical teams in the acute setting and assess evidence for any adverse consequences to patient safety or healthcare delivery.

**Design:**

Prospective cohort study (1 week) and analysis of linked electronic health records (3 years).

**Setting:**

UK tertiary care centre.

**Participants:**

All patients admitted sequentially to the acute medical service under an infectious diseases acute physician (IDP) and other medical teams during 1 week in 2013 (n=297), and 3 years 2012–2014 (n=47 585).

**Primary outcome measure:**

Antibiotic use in days of therapy (DOT): raw group metrics and regression analysis adjusted for case mix.

**Secondary outcome measures:**

30-day all-cause mortality, treatment failure and length of stay.

**Results:**

Antibiotic use was 173 vs 282 DOT/100 admissions in the IDP versus non-IDP group. Using case mix-adjusted zero-inflated Poisson regression, IDP patients were significantly less likely to receive an antibiotic (adjusted OR=0.25 (95% CI 0.07 to 0.84), p=0.03) and received shorter courses (adjusted rate ratio (RR)=0.71 (95% CI 0.54 to 0.93), p=0.01). Clinically stable IDP patients of uncertain diagnosis were more likely to have antibiotics held (87% vs 55%; p=0.02). There was no significant difference in treatment failure or mortality (adjusted p>0.5; also in the 3-year data set), but IDP patients were more likely to be admitted overnight (adjusted OR=3.53 (95% CI 1.24 to 10.03), p=0.03) and have longer length of stay (adjusted RR=1.19 (95% CI 1.05 to 1.36), p=0.007).

**Conclusions:**

The IDP-led group used 30% less antibiotic therapy with no adverse clinical outcome, suggesting antibiotic use can be reduced safely in the acute setting. This may be achieved in part by holding antibiotics and admitting the patient for observation rather than prescribing, which has implications for costs and hospital occupancy. More information is needed to indicate whether any such longer admission will increase or decrease risk of antibiotic-resistant infections.

**Strengths and limitations of this study**

- Clinical relevance of study population, conducted in ‘real-life’ hospital environment.
- Robust risk-adjusted multivariable analysis of prescribing to show differences in antibiotic use is not an artefact of case mix.
- Analysis of clinical outcome as well as prescribing outcome showing antibiotic reduction was achieved safely.
- Data collection by single physician unblinded to management team, possibility of recording bias, though objective criteria used where possible.
- Single-centre study; relevance to other institutions requires further exploration.

## Introduction

Owing to the increasing profile of the threat of antimicrobial resistance, there has been an increased focus on measuring and reducing antibiotic use.^1^ Associations between antibiotic consumption and resistance^2^ suggest that reducing antibiotic use will reduce selection for resistance. Hospital antibiotic use accounts for 15% of antibiotic use, and up to 65% of broad-spectrum antibiotic use in England.^3–5^ Reducing antibiotic use in the acute hospital setting provides unique challenges, requiring rational decision-making before full results of diagnostic tests are available, with uncertainty over diagnosis and severity of illness, and in a pressured environment. Any pressures to reduce antibiotic prescribing have to be balanced with initiatives, such as the Surviving Sepsis Campaign, which highlight the importance of not delaying antibiotics on patient outcome.

Previous studies have shown that management input from an infectious diseases acute physician (IDP) can reduce antibiotic use in the acute hospital setting. Studies have demonstrated that IDP input for patients managed under other acute physicians is associated with shorter durations of treatment, with more patients having treatment stopped or shortened.^6^ IDP input has also been associated with reduced length of stay,^7^ costs of antibiotic therapy^8^ ^9^ and improved clinical outcome 8 9(also reviewed in).^10^ ^11^

A few studies have compared management of acute medical patients by infectious disease and non-infectious disease-trained hospitalists.^12^ ^13^ Eron *et al*^13^ looked retrospectively at the management of patients with diagnosed infections, showing shorter length of stay and better patient satisfaction, but did not examine quantity of antibiotic use,^13^ while Borer *et al*^12^ compared management of febrile syndromes between patients managed by IDP hospitalists and non-IDP hospitalists, and showed improved antimicrobial appropriateness and accuracy of diagnosis, but did not examine length of stay or mortality.^12^

Prescribing quality assessments in the acute medical setting have suggested that up to one-third of prescriptions may be inappropriate.^14^ ^15^ However, comprehensive assessments of prescribing interventions highlight the lack of reporting of clinical outcome, where antibiotic reductions have been demonstrated.^16^ ^17^ With an onus on all physicians to reduce antibiotic use, it is desirable to examine further what strategies IDPs may be using to reduce antibiotic use that can be subsequently employed by general medical physicians themselves. Evidence is also required that these reductions can be achieved safely, in the real-life hospital environment, with an acutely unwell, heterogeneous group of patients.

The goal of our study was to identify whether general medical physicians could make significant, safe antibiotic reductions, and to assess the magnitude of this achievable reduction in an entire, mixed patient cohort from point of referral to the acute medical service, rather than in selected conditions.

We also wished to investigate whether there was any evidence for adverse effects from lower antibiotic use on patient outcome. We were able to do this because in our institution, a tertiary centre, some acute medical unit teams are led by an IDP. Our primary objective was therefore to assess the difference in total amount of antibiotic used between IDP-led and non-IDP-led teams. Comparison at a consultant team level reflected working practices, and previous studies supporting the commonly held view among physicians that it is the ‘senior doctor who ultimately decides what antimicrobial is prescribed’.^18^ Our secondary objectives were to assess whether there was any evidence that differential antibiotic use led to adverse clinical outcomes or increased length of stay in the different teams, and to identify specific differences in antibiotic prescribing strategies. Antibiotic and clinical data were collected prospectively as part of a benchmarking audit, and an exploratory post-hoc analysis of prescribing differences between IDP and non-IDP teams was performed. To further investigate outcome between IDP and non-IDP management with greater statistical power, a 3-year data set of routine electronic health records was also analysed for mortality and length of stay (although patient-level prescribing and clinical response data were not available in this data set).

## Methods

### Clinical and prescribing data

Antibiotic prescribing data were collected prospectively on all acute admissions to the medical service in a UK tertiary care centre (John Radcliffe Hospital, Oxford, from 08:00 16 April to 08:00 23 April 2013) by the lead author, an active physician in the department, but not working during this period. Data were collected as a benchmarking audit of antimicrobial prescribing. In this service, patients are admitted from primary care and Emergency Department referrals, and managed under an on-call consultant acute physician and their team, who share an on-call team rota with three shifts per day (08:00–15:59, 16:00–20:59, night shift 21:00–07:59). The team makes initial management decisions (including antibiotic prescribing) before or during a consultant ward round of all newly admitted patients. After initial care, a small proportion of patients are transferred for operational reasons to the nearest available bed under any team or to other non-acute specialities (<5%); the majority are managed under admitting team (patient flow chart: see online supplementary figure S1). The acute team usually consists of a consultant physician (senior), and three physicians in training with 1–7 years of postqualification experience (juniors). An IDP with triple accreditation in infectious diseases, microbiology and general (internal) medicine performed usual clinical duties during the audit period. Acute physicians were informed antibiotic prescribing audits were ongoing, but were not aware of exact audit dates.

10.1136/bmjopen-2015-010969.supp2 Supplementary figures

All patients who had a documented referral to medical team and were reviewed by them were included, even if they were sent home from the emergency department without attending a medical ward. Data were collected on antibiotic use, managing team, principal diagnosis and factors affecting antibiotic prescribing including admission timing, allergies and comorbid status. Principal diagnosis was copied from the attending physician, not made retrospectively. Infection likelihood was graded by the lead author based on the consultant physician's documented review using the objective criteria: high likelihood—most likely diagnosis documented was infection; medium—infection was among many differential diagnoses documented; and low—list of differential documented diagnoses did not include infection. Compliance with local empirical treatment guidelines was also recorded. First antibiotic was compared to the guidance for the documented working diagnosis and judged inappropriate if it did not match. Appropriateness of duration was assessed for completed courses of empirical therapy without documented complications or changes in diagnosis. Clinical outcomes collected were mortality (30 days post discharge), readmission, escalation/restarting antibiotic treatment and disease-related complications (see online supplementary information table S1). A composite clinical outcome of ‘treatment failure’ (any of these outcomes) was also considered. Delays in treating patients with sepsis were also recorded. Data were extracted by review of drug chart, clinical and electronic notes by the lead author.

10.1136/bmjopen-2015-010969.supp1 Supplementary tables

A larger 3-year data set of hospital electronic health records was also extracted from the Infections in Oxfordshire Research Database, for all patients admitted under the acute medicine service from 00:01 on 1 January 2012 to 23:59 on 31 December 2014, including managing consultant, International Classification of Diseases, Revision 10 (ICD-10) diagnosis codes, length of stay, mortality and readmissions. Patient-level prescribing and clinical response data were not available in this data set. During this period, the IDP performed clinical duties in the acute medicine service, and other departments together with periods of leave, thus admitting ∼1% of patients in this data set.

Antibiotic use was calculated using several metrics using previously published definitions,^19^ and classified according to physician team who managed the patient at admission, and included antibiotics to be taken postdischarge and antibiotics used if transferred to non-acute medical specialties (including intensive care units). Bed-days were calculated from changes in calendar date, counting patients admitted and discharged on the same day as 1 day. The treatment-related length of stay was duration until recorded as ‘medically fit for discharge’. The primary outcome metric of days of therapy (DOT) per patient admission was chosen as it best reflected physician prescribing decision-making and had fewer potential confounding factors, namely local formulary choice mismatches with the WHO-standard defined daily dose (DDD)^20^ and changes in length of stay; it is a metric choice supported by other prescribing analyses using regression modelling.^19^ ^20^ DOT counts each antibiotic received for 1 day as 1 DOT (so 2 antibiotics for 1 day=2 DOT): we also considered length of therapy (LOT: total calendar days on which one or more antibiotics were taken).

### Statistical analysis

We hypothesised, based on small pilot data, that IDP management would lead to 30% less total antibiotic use, with no difference in clinical adverse composite outcome. In total, 367 patients provided at least 80% power to detect a reduction in overall antibiotic use from 300 to 210 DOT/100 admissions (30% reduction) based on 10 000 simulations from a Poisson distribution, assuming that 20% patients were admitted under IDP during audit period, overall 30% patients received antibiotics and two-sided α=0.05. Acute medicine admits 300–400 patients per week; 1 week was therefore chosen as a representative audit sample including out-of-hours and weekend prescribing.

Baseline characteristics between IDP and non-IDP groups were compared using Wilcoxon Rank Sum tests for continuous and Pearson χ^2^ or Fisher's exact tests for categorical factors. Antibiotic prescribing was modelled as a two-step process (initiation and subsequent decision about total quantity), representing clinical experience and national prescribing guidance,^21^ using a zero-inflated Poisson model. Because there was clear separation between DOT in those receiving antibiotics and the zero DOT in those not receiving antibiotics, and because we were concerned about the possibility of non-linear effects of some continuous predictors (particularly age and laboratory test results), predictors of receiving an antibiotic prescription or not were first identified using multivariable fractional polynomial logistic regression with backwards elimination (exit p=0.05; Stata mfp; based on the 20 factors in table 1). Factors retained in this model were included in the zero-probability component of a zero-inflated Poisson regression, with predictors of antibiotic quantity identified using a second phase of backwards elimination (exit p=0.05). In regression models, DOT and length of stay were truncated at their 95th percentiles (18 and 17 respectively) to avoid undue influence from outlying observations leading to overfitting. A small number of patients did not have any biomarkers of infection measured (19/297); only available data were used in the regression analysis (complete case). Risk-adjusted 30-day mortality was assessed using a multivariable fractional polynomial logistic model (backward elimination, exit p=0.05). This analysis was repeated in a larger 3-year data set of 47 585 admissions to acute medicine 2012–2014 (90% power to detect an IDP-associated mortality increase from 6% to 11%, assuming IDP saw 1% admissions). Analysis was performed using Stata V.13.1.

**Table 1:** Patient characteristics at presentation to the acute medical service

	Non-IDP (N=241)	IDP (N=56)	
Characteristics	N (%) or median (IQR)	N (%) or median (IQR)	p Value
Age	72 (52–82)	79 (62–83)	0.15
Female	137 (57)	30 (54)	0.66
Charlson score	1 (1–2)	1 (1–2)	0.97
Ischaemic heart disease	34 (14%)	6 (11%)	0.50
Congestive cardiac failure	18 (7%)	5 (9%)	0.71
Pulmonary disease	64 (27%)	10 (18%)	0.18
Cerebrovascular disease	24 (10%)	3 (5%)	0.44
Dementia	13 (5%)	7 (12%)	0.06
Renal disease	25 (10%)	6 (11%)	0.94
Liver disease	5 (2%)	0 (0%)	0.59
Cancer	19 (8%)	5 (9%)	0.80
Immunosuppression*	7 (3%)	4 (7%)	0.13
Sepsis†	20 (8%)	5 (9%)	0.88
Community antibiotic therapy	39 (16%)	11 (20%)	0.53
Fever on admission	17 (7%)	5 (9%)	0.63
C reactive protein	14 (3–60)	14 (5–70)	0.94
White cell count (10^9/l)	8 (7–11)	8 (6–10)	0.03
Weekend admission	57 (24%)	9 (16%)	0.22
Night-time admission	58 (24%)	17 (30%)	0.33
Admission with frailty syndrome‡	28 (12%)	12 (21%)	0.05

p Values calculated using Wilcoxon rank sum tests for continuous variables and χ^2^ tests for categorical variables, unless any cell number was <5 or cell percentage <5% in which case Fisher’s exact test was used.

*Immunosuppression defined as HIV infection, haematological malignancy, other malignancy, renal failure, previous transplant, chemo/radiotherapy in past 5 years, immunosuppressive drugs, systemic corticosteroids (0.5 mg/kg/day for >14 days). †Sepsis defined as per the American Society of Critical Care Medicine as fulfilling Systemic Inflammatory Response Syndrome (SIRS) criterion of 2 or more of: respiratory rate >20, pulse >90, leucocytes <4 or >12, temperature <36°C or >38°C with an infective source.

‡Frailty syndrome as defined by the British Geriatric Society^22^—falls, immobility, delirium/dementia, polypharmacy, incontinence, end-of-life care.

IDP, infectious diseases physician.

### Ethics

The baseline antibiotic prescribing audit was approved by the Oxford University Hospitals Clinical Audit Service. Mortality and admission comparisons over 2012–2014 were an approved analysis within the Infections in Oxfordshire Research Database, which has Research Ethics Committee (14/SC/1069) and UK Health Research Authority (5-07(a)/2009) approval as an anonymised database without individual informed consent. The IDP gave consent to be identified in these data, and for the prescribing outcome analysis to be performed.

## Results

### Patients managed under IDP received ∼30% less antibiotic overall, due to fewer patients being given antibiotics, and shorter antibiotic courses

During a 1-week period, the IDP-led team admitted 56 patients, and non-IDP Acute Physician teams admitted 241 patients. Teams admitted similar patients, except IDP patients had slightly lower white cell counts (WCC) and were more likely to be admitted with a frailty syndrome (table 1). Group metrics showed the IDP team used 173 DOT per 100 admissions versus 282 DOT per 100 admissions in the non-IDP group, an (unadjusted) reduction of 39% (table 2). A similar percentage reduction was seen in all metrics assessed. 14/56 (25%) IDP patients were given an antibiotic versus 85/241 (35%) non-IDP patients.

**Table 2:** Antibiotic use according to managing physician in a range of metrics

Metric					
Group metrics	Non-IDP (241)	IDP (56)	All	Unadjusted RR (95% CI)	p Value
Days of therapy (DOT)/100 admissions	282	173	261	0.61 (0.50 to 0.76)	<0.001
Defined daily doses (DDDs)/100 admissions	340	227	320	0.67 (0.55 to 0.80)	<0.001
Broad-spectrum DOT/100 admissions	169	98	156	0.58 (0.44 to 0.77)	<0.001
Length of therapy (LOT)/100 admissions	210	129	195	0.61 (0.48 to 0.78)	<0.001
DDDs/100 bed days	66	38	60	0.47 (0.31 to 0.70)	<0.001
Patient-level metrics	n (%) or med (IQR)	n (%) or med (IQR)		Adjusted OR /RR (95% CI)	p Value
Given an antibiotic*	85 (35%)	14 (25%)	99 (33%)	0.25 (0.07 to 0.84)	0.03*
DOT†	0 (0–5)	0 (0–0.5)	0 (0–5)	0.71 (0.54 to 0.93)	0.01†
DDDs†	0 (0–5.25)	0 (0–0.13)	0 (0–4.5)	0.67 (0.53 to 0.90)	0.006 †
LOT†	0 (0–5)	0 (0–0.5)	0 (0–3)	0.69 (0.52 to 0.94)	0.02†
DOT if started on an antibiotic	6 (5–9)	6 (3–7)	6 (5–9)	–	–
LOT if started on an antibiotic	5 (4–7)	4 (2–6.5)	5 (3–7)	–	–
Broad-spectrum*,‡ antibiotic given	70 (29%)	11 (20%)	81 (27%)	0.53 (0.19 to 1.52)	0.24*
Broad-spectrum antibiotic DOT†	0 (0–2)	0 (0–0)	0 (0–1)	0.75 (0.54 to 1.03)	0.08†
Broad:narrow ratio	408/271 (1.51:1)	55/42 (1.31:1)	463/313 (1.48:1)	–	–
Given intravenous antibiotic*	52 (22%)	8 (14%)	60 (20%)	0.39 (0.12 to 1.27)	0.12*
Intravenous DOT†	0 (0–0)	0 (0–0)	0 (0–0)	0.76 (0.49 to 1.18)	0.23†
Intravenous DOT if given intravenous	2 (1–3)	1 (1–4.5)	2 (1–4)	–	–

*Multivariable logistic regression model based on backwards elimination from IDP plus table 1 factors.

†From multivariable zero-inflated Poisson regression models based on backwards elimination from IDP plus table 1 factors (see Methods). Table 3 presents the full multivariable model for DOT; predictors were similar for DDDs and LOT with the only differences being that an admission at the weekend was additionally associated with greater DDDs and Charlson score was not associated with DDDs; and the only factors associated with greater LOT were male sex, sepsis and higher CRP on admission. Predictors for giving a broad spectrum antibiotic and being given an intravenous antibiotic were immunosuppression, higher CRP and WCC on admission, with predictors of higher broad spectrum DOT being fever, higher CRP, female sex, age, deprivation index and admission at night, and predictors of greater intravenous DOT were sepsis, higher CRP, deprivation index and female sex.

‡Broad-spectrum antibiotics: co-amoxiclav, ceftriaxone, meropenem, gentamicin, clindamycin, ceftazidime, ciprofloxacin, piperacillin/tazobactram. CRP, C reactive protein; DDD, defined daily dose; DOT, days of therapy; IDP, Infectious Diseases Physician; LOT, length of therapy; RR, rate ratio; WCC, white cell counts.

**Table 3:** Independent predictors of days on therapy (DOT) in a zero-inflated Poisson model

	Factor	Adjusted OR (95% CI)	p Value
Association with starting an antibiotic	Management under IDP	0.32 (0.10 to 0.99)	0.05
	Presentation with frailty syndrome	3.87 (1.41 to 10.67)	0.01
	Received community antibiotics	10.57 (3.91 to 28.60)	<0.001
	CRP on admission (per 1 mg/dL higher)	1.02 (1.02 to 1.03)	<0.001
	Immunosuppression	49.12 (2.85 to 847.89)	0.007
	White cell count on admission (per 1^9/L higher)	1.22 (1.11 to 1.34)	<0.001
	**Factor**	**Adjusted RR (95% CI)**	
Association with greater days on therapy (DOT) per admission	Management under IDP	0.71 (0.54 to 0.93)	0.01
	Female	0.77 (0.65 to 0.92)	0.003
	Age (per 10 years older)	1.08 (1.02 to 1.13)	0.005
	Charlson score (per 1 unit higher)	0.94 (0.90 to 0.99)	0.01
	Sepsis	1.38 (1.15 to 1.65)	0.001
	Night admission	1.40 (1.18 to 1.67)	<0.001
	CRP on admission (per 10 mg/dL higher)	1.02 (1.00 to 1.03)	0.02
	Presentation with frailty syndrome	0.77 (0.62 to 0.97)	0.02

Sepsis and fever perfectly predicted antibiotic use and so therefore could not be included in the zero-component of the model. No other factors in table 1 were independently associated.

CRP, C reactive protein; IDP, infectious diseases physician; RR, rate ratio.

### Multivariate analysis adjusting for patient case mix confirms IDP management associated with lower antibiotic use

In a patient-level risk-adjusted model (see Methods, table 3), IDP management was associated with a significantly lower odds of initiating antibiotics (adjusted p=0.03) and fewer DOT (p=0.01) suggesting that the lower antibiotic use in the IDP group was not simply an artefact of case mix. Patients presenting with sepsis or fever, immunosuppressed or frail patients, those with higher C reactive protein (CRP) or WCC at admission, and those who had received community antibiotic therapy were more likely to start antibiotics in hospital (table 3). Factors associated with greater DOT were male sex, older age, sepsis, higher CRP on admission, lower Charlson score^23^ and admission during the night.

### No evidence of differing antibiotic spectrum, route, choice, diagnosis treated or guideline compliance between non-IDP and IDP teams

There was no evidence of a difference in number of patients started on a broad-spectrum antibiotic (adjusted p=0.24), broad-spectrum antibiotic DOT (p=0.08), number of patients started on an intravenous antibiotic (p=0.12) or intravenous DOT (p=0.23). The reduction in total antibiotic use was not due to reduced use of antibiotic combinations; analysis by total length of therapy (which counts days on single or combination therapy as a single day) also showed a 39% unadjusted total reduction (129 vs 210 LOT/100 admissions) (table 2). Antibiotic choice and diagnosis treated were similar between groups (see online supplementary information table S2 and supplementary figure S2). There was no evidence of differences between IDP and non-IDP groups in compliance with guidelines for first prescription (14/14 (100%) vs 78/85 (92%) prescriptions compliant, respectively, p=0.59), compliance with guideline duration for uncomplicated infection without change in diagnosis (4/5 (80%) vs 39/44 (89%) completed courses compliant, respectively, p=0.58) or adherence to documenting a postprescription review (9/12 (75%) having a documented review in the IDP group and two decisions to stop (22%), compared with 47/62 (76%) reviews in the non-IDP group, with 11 decisions to stop (21%) p>0.9). As numbers were small, data were insufficient to conclude that prescribing quality was similar between IDP and non-IDP teams, but there was no signal that the significant differences observed in the total volume of antibiotics prescribed could be clearly attributed to these factors.

### A ‘hold-and-observe’ strategy in stable patients where the diagnosis is not established may contribute to reduced IDP antibiotic use

There was no difference in antibiotic prescription rates between IDP versus non-IDP groups in perceived high-risk patients (classified according to presenting features as having a high likelihood of infection, sepsis or immunosuppression), or in patients with low likelihood of infection. However, there was a difference in antibiotic use in cases of uncertain infective diagnosis without adverse features (table 4). In the IDP group, 2/16 (13%) were started on antibiotics on admission versus 31/69 (45%) in the non-IDP group (p=0.02). About 2/16 patients in the IDP group who had antibiotics initially held subsequently had antibiotics started for the presenting issue after a period of observation, one for possible bronchitis and one for a possible urinary tract infection in the absence of conclusive culture result. Given the difference in prescribing in patients of uncertain diagnosis, we also looked specifically at this group and found no evidence of difference in clinical outcome (table 5).

**Table 4:** Likelihood of starting antibiotics on day of admission in IDP and non-IDP teams, by likelihood of infection and risk of delaying antibiotics

Likelihood of infection as per documented review*	Risk of delaying antibiotics (presence of sepsis/immunosuppression)	Non-IDP (N=241) Number started on antibiotics on day of admission/total (%)	IDP Number started on antibiotics on day of admission/total (%)	p Value†
High	High	17/17 (100)	5/5 (100)	–
	Low	22/27 (81)	4/5 (80)	1.00
Medium	High	7/9 (78)	1/1 (100)	1.00
	Low	31/69 (45)	2/16 (13)	0.02
Low	High	0/0 (0)	0/0 (0)	–
	Low	1/119 (1)	0/29 (0)	1.00

*Based on documented consultant review: high=infection most likely diagnosis; medium=infection on list of differential diagnoses; low=infection not on list of differential diagnosis.

†Fisher’s exact test.

IDP, infectious diseases physician.

**Table 5:** Clinical outcomes including mortality, complications and admissions over 1 week audit and 3 years administrative data

Clinical outcome One week audit: all patients	Non-IDP n (%) or median (mean) (IQR)	IDP n (%) or median (mean) (IQR)	Total n (%) or median (mean) (IQR)	Adjusted OR/RR* (95% CI)	
	(n=241)	(n=56)	(n=297)		p Value*
30-day mortality	36 (15%)	7 (13%)	43 (15%)	1.36 (0.53 to 3.54)	0.52
Escalation of therapy	12 (5%)	3 (5%)	15 (5%)		
Disease-specific complications	2 (1%)	0 (0%)	2 (1%)		
ICU admission	4 (2%)	2 (4%)	6 (2%)		
Readmission	50 (21%)	10 (18%)	60 (20%)		
Composite outcome	87 (36%)	19 (34%)	106 (36%)	1.21 (0.60 to 2.48)	0.59
Admitted overnight	168 (70%)	49 (88%)	217 (73%)	3.53 (1.24 to 10.03)	0.03
Length of stay	2 (5.2) (2–6)	4 (5.9) (3–6)	3 (5.3) (2–6)	1.19 (1.05 to 1.36)	0.007
Treatment-related length of stay	2 (4.2) (2–5)	3 (5.0) (2–6)	3 (4.3) (2–5)	1.27 (1.11 to 1.47)	0.001
One week audit: patients of uncertain diagnosis	(n=78)	(n=17)	(n=95)		
30-day mortality	11 (14%)	3 (18%)	14 (15%)	1.16 (0.26 to 5.17)	0.85
Composite outcome	31 (40%)	6 (35%)	37 (39%)	0.99 (0.29 to 3.45)	0.99
Administrative data, 1 Jan 2012–31 Dec 2014	(n=47 108)	(n=477)	(n=47 585)		
30 day mortality	2987 (6.3%)	27 (5.7%)	3014 (6.3%)	0.92 (0.62 to 1.37)	0.68
Admitted overnight	37 329 (79%)	397 (83%)	37 726 (79%)	1.55 (1.20 to 2.01)	0.001
Length of stay	3 (6.8) (2–8)	4 (7.7) (2–8)	3 (6.8) (2–8)	1.15 (1.11 to 1.19)	<0.001

*Multivariable regression: 1-week data set: C reactive protein (CRP), white cell count (WCC) and Charlson score independently associated with 30-day mortality. Sepsis, Charlson score and admission with a frailty syndrome independently associated with adverse composite outcome. Charlson score, age and later time of day independently associated with admission. Charlson score, age, presentation with a frailty syndrome, community antibiotics, WCC and admission during a weekday independently associated with longer length of stay. Three-year data set: Charlson score, age, male gender and non-weekday admission independently associated with mortality, Charlson score, age hour of admission and non-weekday admission independently associated with admission overnight, Charlson score, female, age, hour of admission and non-weekday admission independently associated with longer length of stay.

ICU, intensive care unit; IDP, infectious diseases physician; RR, rate ratio.

### IDP management was not associated with delays in treatment, increased mortality or treatment failure, but was associated with longer length of stay

There was no evidence that management under IDP was associated with risk-adjusted 30-day all-cause mortality or the composite ‘treatment failure’ outcome (adjusted p>0.5; table 5). However, with relatively small numbers, 95% CIs were wide. There was no evidence of delays in treating sepsis, with 11/20 non-IDP (55%) and 4/5 (80%) IDP patients presenting with sepsis based on objective criteria^24^ receiving antibiotics in <1 hour (p=0.62).

However, IDP management was associated with a slight, though significant, increase in length of stay (adjusted p=0.007; table 5). One of the main contributors was an increased odds of overnight admission in the IDP group, with 49/56 (88%) patients admitted overnight versus 168/241 (70%) non-IDP patients (adjusted p=0.03). This was consistent with the previously noted ‘hold and admit’ strategy of the IDP, versus a shorter length of stay ‘prescribe and discharge’ strategy in non-IDP physicians.

To investigate clinical outcome associated with IDP management further, and explore whether these findings could be replicated in a larger data set, we also looked in a 3-year administrative data set of over 47 000 admissions (2012–2014). Similar to our 1-week data set, this comprised patients admitted to the acute, unselected medical take, during which time the same consultant teams were operating with broadly the same antibiotic policy. Patient characteristics available in the 1 week and 3-year data sets were broadly similar in terms of age, gender, Charlson score (as calculated using hospital ICD-10 coding in the 3-year data set) and admission at a weekend (see online supplementary information table S3).

There was no evidence of IDP-associated excess mortality risk in this much larger data set (adjusted p=0.68; table 5; full regression model in online supplementary information table S4). Similarly to the 1-week data set, IDP management was associated with increased likelihood of overnight admission and longer length of stay (adjusted p<0.001). There was no evidence that the relationship between IDP management and these clinical outcomes depended on age, Charlson score or time/date of admission (heterogeneity/interaction p>0.05). While prescribing and patient clinical data were not available in this larger data set, these findings increased our power to detect differences in clinical outcome, and suggest the increased likelihood of admission and longer length of stay seen with IDP management were not restricted to our smaller 1-week data set.

## Discussion

This analysis of antibiotic prescribing data from a benchmarking audit suggests that IDP management was associated with ∼30% less total antibiotic use in acute medical admissions compared with other acute physicians, with fewer patients being given an antibiotic and shorter courses. Examination of clinical outcome in these audit patients, and in a much larger hospital administrative data set, suggests this antibiotic reduction was achieved without adverse patient outcome. The reduction in total antimicrobial use by the IDP was not due to differences in antibiotic choice or compliance with guidance. A key contributory factor was reduced antibiotic initiation in stable patients where there was diagnostic uncertainty. However, as a potential unintended consequence, there was an association between IDP management and increased overnight admissions and length of stay. In the IDP team, the strategy therefore appeared to be ‘hold and observe’, perhaps where a perceived antibiotic ‘safety net’ was replaced with one based on observation and greater degree of diagnostic certainty. This was supported by anecdotal feedback from junior physicians, who identified (1) an attitude in the IDP team of ‘you must justify why you gave antibiotics’ compared with ‘give antibiotics, just in case’ (as previously described by others),^25^ (2) the use of antibiotics to ‘give a diagnosis’ in cases of uncertainty and (3) the view that antibiotics are often used as a ‘safety net’ where there is pressure from patients, healthcare targets^26^ or limited bed availability to avoid admission. While a full investigation of these factors was outside the remit of this study, it reinforces the importance of the complex decision-making process and social factors on antibiotic prescribing.^18^ Increased admissions may also reflect a tendency to pursue greater diagnostic precision for infection diagnoses, as previously noted by Borer *et al*.^12^ Alternatively, physicians trained in other specialties such as elderly care may have more experience with community care resources, enabling prompt discharge of the predominantly older medical population, though this would not explain the discrepancy in antibiotic use since antibiotic treatment after discharge was taken into account when determining DOT.

Study strengths include the ‘real life’ clinical relevance of the study population and robust risk-adjusted multivariable analysis of patient-level data to study prescribing and clinical outcome. Limitations include the restricted audit size, which was also smaller than planned. While we were able to subsequently examine a larger administrative data set with similar patient characteristics to assess outcome, individual-level prescribing and clinical data were not available, requiring extrapolation that IDP and non-IDP antibiotic use would have been similar over the longer period of time. During the audit, data collection was by a single clinician unblinded to management team, with possibility of recording bias for clinical factors, although objective criteria were used wherever possible. Although acute physicians were made aware of the audit and the exact date were not announced, the IDP may have been more aware of the monitoring due to closer professional relationships with the lead author, and larger differences in prescribing found due to extra attention on antibiotic use in the IDP team. The study focused on one IDP; while IDPs with similar training and integration to acute medicine exist around the UK, replicating the findings with more IDPs at different locations is required before these findings can be generalised to IDPs as a group. The study was single centre; understanding generalisability to other centres remains important. Cost impact has not been assessed, though we note that as overnight stays currently cost over £250,^27^ even small increases in admissions will likely dwarf modest antibiotic cost savings. Our evaluation did not challenge the validity of the documented working diagnosis, which may have been influenced by preferred antibiotic choice. Judgements on appropriateness can differ substantially depending on the assessor;^28^ the resource requirements for robust assessment were outside those available for this study. Future work should include investigating whether the relationship between reduced antibiotic use and more admissions is seen in multiple different healthcare settings, and use of newly available larger hospital data sets with electronic prescribing to further elucidate patient and procedural consequences of changes in prescribing strategies. Ultimately, more information regarding the complex factors contributing to the risk and spread of antimicrobial resistance in a population is urgently needed.

Our findings confirm those of other studies of the potential for antibiotic use reduction in the acute mixed-case setting. A study of 129 adult patients admitted to a US tertiary hospital found 30% of total antimicrobial days were judged unnecessary by study investigators,^15^ but there was no intervention to treat patients with this reduced amount. Another interventional study found that implementing a post-prescription review in 5 US academic tertiary care centres and 2428 acute hospital patients found 7% and 16% reductions in antibiotic use at two sites with established stewardship programmes, but no reduction at the other three sites.^21^ This study did not include antibiotics prescribed for use after discharge and did not examine clinical outcomes. Analysis of antibiotic use in a risk-adjusted analysis in 103 US academic medical centres at an institutional level showed significant interhospital variability in expected antibiotic use.^29^

imilar to previous studies, we found IDP input associated with a reduction in antibiotic use.^10^ ^13^ However, the increased length of stay we observed is contrary to Borer *et al* who found IDP management was associated with shorter lengths of stay.^13^ This study retrospectively assessed management of patients diagnosed with infections, and thus may not have captured management decisions made with acute patients of uncertain diagnosis, which is where we saw the ‘admit and observe’ behaviour which was likely contributory to our IDP-associated longer lengths of stay.

Our study adds to this body of work by demonstrating reduced antibiotic prescribing using patient-level data to adjust for case mix and conducting a robust analysis of clinical outcome data to assess patient safety, providing further evidence of the potential for safe antibiotic reduction. It also examines for the first time the effects of IDP management in patients at presentation, rather than after a period of assessment and provisional diagnosis of infection being made. It reports an association with reduced antibiotic use and increased admissions in the acute setting, contrary to previous studies.^7^ ^13^

In conclusion, our study provides further support that antibiotic reduction can be achieved safely in the acute setting, but at the expense of more and longer admissions. This could be done by replacing a perceived ‘antibiotic safety net’ (prescribe and discharge) with one based on observation and greater degree of diagnostic certainty (hold and observe). While the findings require replication in other healthcare settings, they suggest that attempts to reduce antibiotic use may come at the expense of increasing admissions and exposure to the hospital environment; this may increase costs to healthcare services and the burden on already-stretched acute services. Given that admission to hospital is a risk factor for resistant infection, it may also conversely increase the spread of resistance.^30^ Ultimately, while this study supports the potential to reduce antibiotic use, it also highlights the need for robust assessment of potential ‘unintended consequences’ of antibiotic reduction policies and their effect on clinical, as well as prescribing, outcomes.

## References

[1] Antimicrobial resistance: tackling a crisis for the health and wealth of nations: Review on Antimicrobial Resistance, 2014.

[2] Albrich WC, Monnet DL, Harbarth S Antibiotic selection pressure and resistance in streptococcus pneumoniae and streptococcus pyogenes. Emerg Infect Dis 2004;10:514–17. http://www.ncbi.nlm.nih.gov/pmc/articles/PMC3322805/15109426 10.3201/eid1003.030252 PMC3322805

[3] Cook PP, Catrou PG, Christie JD Reduction in broad-spectrum antimicrobial use associated with no improvement in hospital antibiogram. J Antimicrob Chemother 2004;53:853–9. 10.1093/jac/dkh163 15044426

[4] Cooke J, Stephens P, Ashiru-Oredope D Antibacterial usage in English NHS hospitals as part of a national Antimicrobial Stewardship Programme. Public Health 2014;128:693–7. 10.1016/j.puhe.2014.06.023 25132393

[5] English surveillance programme for antimicrobial utilisation and resistance (ESPAUR): Report Public Health England, 2014.

[6] Lesprit P, Landelle C, Brun-Buisson C Clinical impact of unsolicited post-prescription antibiotic review in surgical and medical wards: a randomized controlled trial. Clin Microbiol Infect 2013;19:E91–7. 10.1111/1469-0691.12062 23153410

[7] Beovic B, Kreft S, Seme K The impact of total control of antibiotic prescribing by infectious disease specialist on antibiotic consumption and cost. J Chemother 2009;21:46–51. 10.1179/joc.2009.21.1.46 19297272

[8] Schmitt S, McQuillen DP, Nahass R Infectious diseases specialty intervention is associated with decreased mortality and lower healthcare costs. Clin Infect Dis 2014;58:22–8. 10.1093/cid/cit610 24072931

[9] Shih CP, Lin YC, Chan YY Employing infectious disease physicians affects clinical and economic outcomes in regional hospitals: evidence from a population-based study. J Microbiol Immunol Infect 2014;47:297–303. 10.1016/j.jmii.2013.01.010 23523046

[10] Pulcini C, Botelho-Nevers E, Dyar OJ The impact of infectious disease specialists on antibiotic prescribing in hospitals. Clin Microbiol Infect 2014;20:963–72. 10.1111/1469-0691.12751 25039787

[11] Kaki R, Elligsen M, Walker S Impact of antimicrobial stewardship in critical care: a systematic review. J Antimicrob Chemother 2011;66:1223–30. 10.1093/jac/dkr137 21460369

[12] Borer A, Gilad J, Meydan N Impact of regular attendance by infectious disease specialists on the management of hospitalised adults with community-acquired febrile syndromes. Clin Microbiol Infect 2004;10:911–16. 10.1111/j.1469-0691.2004.00964.x 15373886

[13] Eron LJ, Passos S Early discharge of infected patients through appropriate antibiotic use. Arch Intern Med 2001;161:61–5. 10.1001/archinte.161.1.61 11146699

[14] Zarb P, Amadeo B, Muller A Identification of targets for quality improvement in antimicrobial prescribing: the web-based ESAC Point Prevalence Survey 2009. J Antimicrob Chemother 2011;66:443–9. 10.1093/jac/dkq430 21084362

[15] Hecker MT, Aron DC, Patel NP Unnecessary use of antimicrobials in hospitalized patients: current patterns of misuse with an emphasis on the antianaerobic spectrum of activity. Arch Intern Med 2003;163:972 10.1001/archinte.163.8.972 12719208

[16] Davey P, Brown E, Charani E Interventions to improve antibiotic prescribing practices for hospital inpatients. Cochrane Database Syst Rev 2013;(4):CD003543 10.1002/14651858.CD003543.pub3 23633313

[17] Dodds Ashley ES, Kaye KS, DePestel DD Antimicrobial stewardship: philosophy versus practice. Clin Infect Dis 2014;59(Suppl 3):S112–21. 10.1093/cid/ciu546 25261538

[18] Charani E, Castro-Sanchez E, Sevdalis N Understanding the determinants of antimicrobial prescribing within hospitals: the role of “prescribing etiquette”. Clin Infect Dis 2013;57:188–96. 10.1093/cid/cit212 23572483 PMC3689346

[19] Ibrahim OM, Polk RE Antimicrobial use metrics and benchmarking to improve stewardship outcomes: methodology, opportunities, and challenges. Infect Dis Clin North Am 2014;28:195–214. 10.1016/j.idc.2014.01.006 24857388

[20] Ibrahim OM, Polk RE Benchmarking antimicrobial drug use in hospitals. Expert Rev Anti Infect Ther 2012;10:445–57. 10.1586/eri.12.18 22512754

[21] Ashiru-Oredope D, Sharland M, Charani E Improving the quality of antibiotic prescribing in the NHS by developing a new Antimicrobial Stewardship Programme: start smart--then focus. J Antimicrob Chemother 2012;67(Suppl 1):i51–63. 10.1093/jac/dks202 22855879

[22] The Silver Book—Quality Care for Older People with Urgent and Emergency Care Needs: The British Geriatric Society, 2012.

[23] Charlson ME, Pompei P, Ales KL A new method of classifying prognostic comorbidity in longitudinal studies: development and validation. J Chronic Dis 1987;40:373–83. 10.1016/0021-9681(87)90171-8 3558716

[24] American College of Chest Physicians/Society of Critical Care Medicine Consensus Conference: definitions for sepsis and organ failure and guidelines for the use of innovative therapies in sepsis. Crit Care Med 1992;20:864–74. 1597042

[25] Cosgrove SE, Seo SK, Bolon MK Evaluation of postprescription review and feedback as a method of promoting rational antimicrobial use: a multicenter intervention. Infect Control Hosp Epidemiol 2012;33:374–80. 10.1086/664771 22418633

[26] Purdy S, Griffin T Reducing hospital admissions. BMJ 2008;336:4–5. 10.1136/bmj.39394.402465.BE 18174566 PMC2174744

[27] Costing patient care, reference costs 2012–13. UK: Department of Health, 2013.

[28] DePestel DD, Eiland EH, Lusardi K Assessing appropriateness of antimicrobial therapy: in the eye of the interpreter. Clin Infect Dis 2014;59(Suppl 3):S154–61. 10.1093/cid/ciu548 25261542

[29] Polk RE, Hohmann SF, Medvedev S Benchmarking risk-adjusted adult antibacterial drug use in 70 US academic medical center hospitals. Clin Infect Dis 2011;53: 1100–10. 10.1093/cid/cir672 21998281

[30] Safdar N, Maki DG The commonality of risk factors for nosocomial colonization and infection with antimicrobial-resistant Staphylococcus aureus, enterococcus, gram-negative bacilli, Clostridium difficile, and Candida. Ann Intern Med 2002;136:834–44. 10.7326/0003-4819-136-11-200206040-00013 12044132

