# Supplementary material for: Antibiotic use and clinical outcomes in the acute setting under management by an infectious diseases acute physician versus other clinical teams: a cohort study
Source: BMJ Open. 2016 Aug 23;6(8):e010969. doi: 10.1136/bmjopen-2015-010969 (PMC5013476; doi:10.1136/bmjopen-2015-010969)

Supplementary Figures

Supplementary Figure 1: Patient admissions and discharges with managing team within the one week benchmarking audit

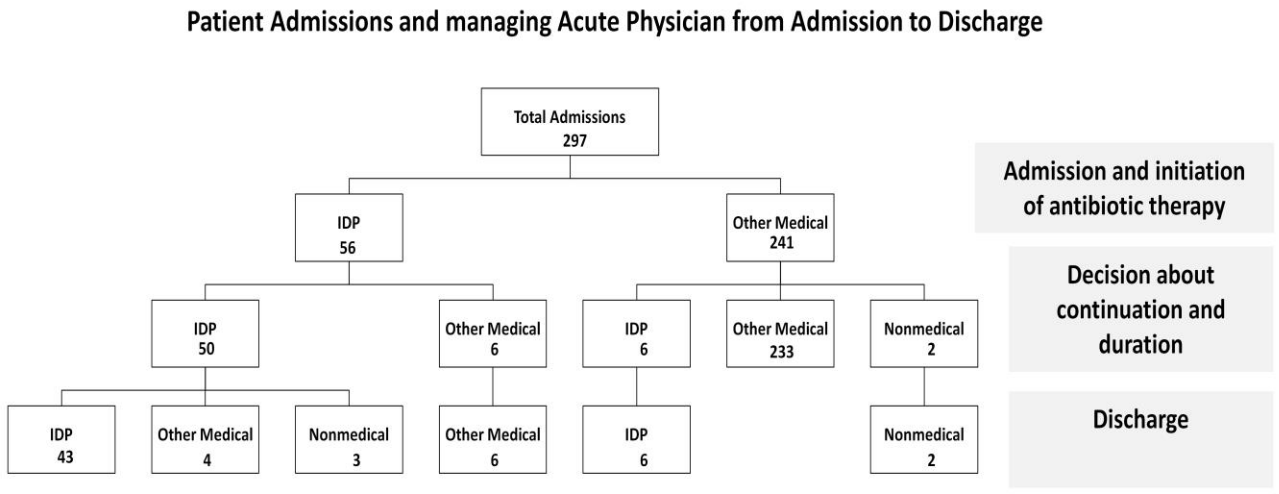

Supplementary Figure 2: Antibiotic use as total days of therapy(DOT), and as a percentage of total DOT used by IDP and Non-IDP groups

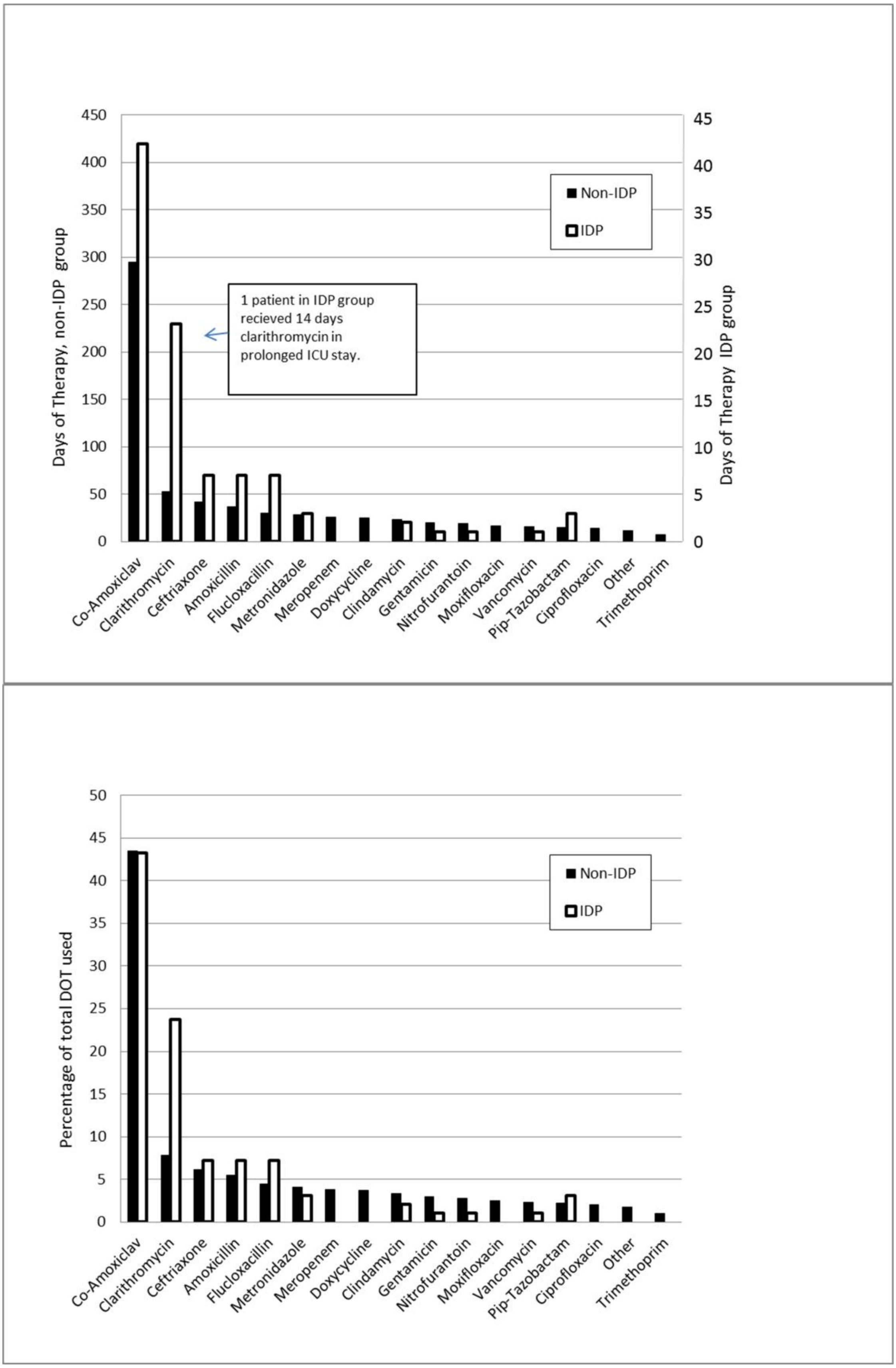

Supplement: Supplementary figures [file bmjopen-2015-010969supp_figures.pdf]
