# Supplementary material for: Antibiotic use and clinical outcomes in the acute setting under management by an infectious diseases acute physician versus other clinical teams: a cohort study
Source: BMJ Open. 2016 Aug 23;6(8):e010969. doi: 10.1136/bmjopen-2015-010969 (PMC5013476; doi:10.1136/bmjopen-2015-010969)
Supplement: Supplementary tables [file bmjopen-2015-010969supp_tables.pdf]

Supplementary Table 1: Disease-Specific Complications collected

Infection Site	Complication
Lower respiratory tract infection or pneumonia or infectious exacerbation of COPD	Empyaema, pleural effusion, abscess
Urinary tract infection	Pyelonephritis
Meningitis	Obstructive hydrocephalus
Cellulitis	Requirement for debridement
Gastrointestinal infection	Perforation, abscess, need for abdominal surgery

COPD=Chronic obstructive pulmonary disease

Supplementary Information Table 2: Working Diagnoses treated and antibiotics used

Working Diagnosis*	Non-IDP(N=85) N(%)	IDP (N=14) N (%)	p**
Chest	36(42)	5(36)	0.64
Urinary tract	14(16)	4(29)	0.28
Unknown	8(9)	0(0)	0.60
Soft tissue	7(8)	1(7)	0.69
Other	6(7)	1(7)	1.00
Meningitis/encephalitis	5(6)	0(0)	1.00
Sepsis	3(4)	2(14)	0.15
Multiple	3(4)	0(0)	1.00
Neutropaenic sepsis	2(0)	0(0)	1.00
Gastrointestinal	1(1)	0(0)	1.00
Antibiotic given			
Co-amoxiclav	56(66)	9(65)	0.91
Clarithromycin	11(13)	4(29)	0.22
Ceftriaxone	16(19)	3(21)	0.73
Amoxicillin	7(8)	1(7)	1.00
Flucloxacillin	5(6)	1(7)	1.00
Meropenem	2(2)	0(0)	1.00
Metronidazole	4(5)	1(7)	0.54
Doxycycline	4(5)	0(0)	0.54
Clindamycin	3(4)	1(7)	0.46
Gentamicin	11(13)	1(7)	1.00
Ciprofloxacin	2(2)	0(0)	1.00
Nitrofurantoin	5(6)	1(7)	1.00
Moxifloxacin	4(5)	0(0)	0.54
Vancomycin	2(2)	1(7)	1.00
Piperacillin-tazobactam	6(7)	1(7)	1.00
Trimethoprim	2(2)	0(0)	0.74
Clindamycin	2(2)	0(0)	0.74
Other	2(2)	0(0)	0.74

**Working Diagnoses: Unknown = older adult with functional decline and chest/urine infection suspected but no clear source localising infection, Other=dental infection, ear infection or antibiotic use for gastrointestinal bleed prophylaxis Multiple = clear localising symptoms/signs for more than one source **calculated using Chi-squared test or Fishers exact test if cell number was <5 or cell percentage <5*

Supplementary information Table 3: A comparison of available patient characteristics in 1 week and 3 year datasets

Characteristics	1 week (N=297)<br>N (%) or med(IQR)	3 year (N= 47585)<br>N (%) or med.(IQR)	p
Age	73 (53-83)	74 (56-84)	0.34
Female	167 (56%)	24775 (52%)	0.16
Charlson score*	1 (0-2)	1 (0-2)	0.33
Weekend admission	66 (22%)	11963 (25%)	0.28

* calculated from hospital ICD-10 coding for the 3 year dataset

Supplementary Information Table 4: Association of factors with in-hospital mortality 1st Jan 2012 – 31st Dec 2014 in 47,585 patients admitted to Acute Medicine.

Risk Factor	OR (95% CI)	P
Management under IDP	0.92 (0.62-1.37)	0.68
Female	0.86 (0.79–0.93)	0.01
Charlson Score (square root transformed: per 1 unit higher)	42.1 (31.7-56.0)	<0.001
Age (per year older)	1.04 (1.04-1.04)	<0.001
Non-weekday-admission	1.16 (1.07-1.26)	<0.001
